# Ho_2_Pd_1.3_Ge_2.7_ – a ternary AlB_2_-type cluster glass system†

**DOI:** 10.1039/d1ra04422b

**Published:** 2021-07-21

**Authors:** Leszek S. Litzbarski, Tomasz Klimczuk, Michał J. Winiarski

**Affiliations:** Faculty of Applied Physics and Mathematics, Advanced Materials Center, Gdansk University of Technology Narutowicza 11/12 80-232 Gdansk Poland leszek.litzbarski@pg.edu.pl

## Abstract

We report a successful synthesis of a ternary AlB_2_-type intermetallic compound. The phase purity was obtained by fine-tuning the Pd : Ge ratio out of the idealized 1 : 3. Attempts to synthesize an Er analogue were not successful. We discuss the instability of the Er analogue based on the atomic size ratio and also suggest that the increased stability of Ho_2_Pd_1+*x*_Ge_3−*x*_ in the Pd-rich range likely stems from a combination of atomic size ratio, electronic, and entropic factors. The new Ho_2_Pd_1.3_Ge_2.7_ compound is found to exhibit cluster glass behavior with a freezing temperature of *T* ≈ 2.3 K.

## Abstract 2

A ternary AlB_2_-type cluster glass system was studied. We discuss the factors responsible for stability of its simple hexagonal structure.

## Graphical abstract

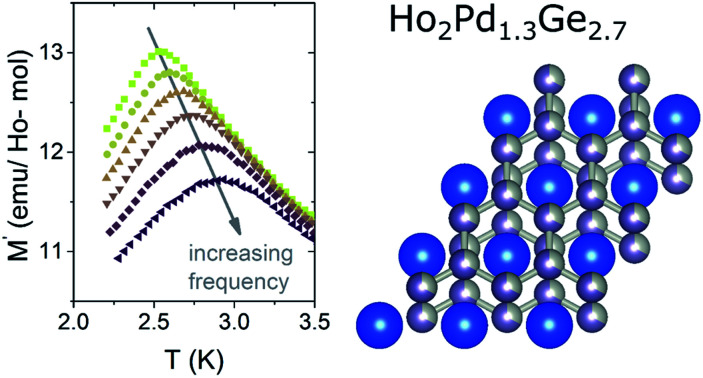

## Introduction

**Fig. 1:**
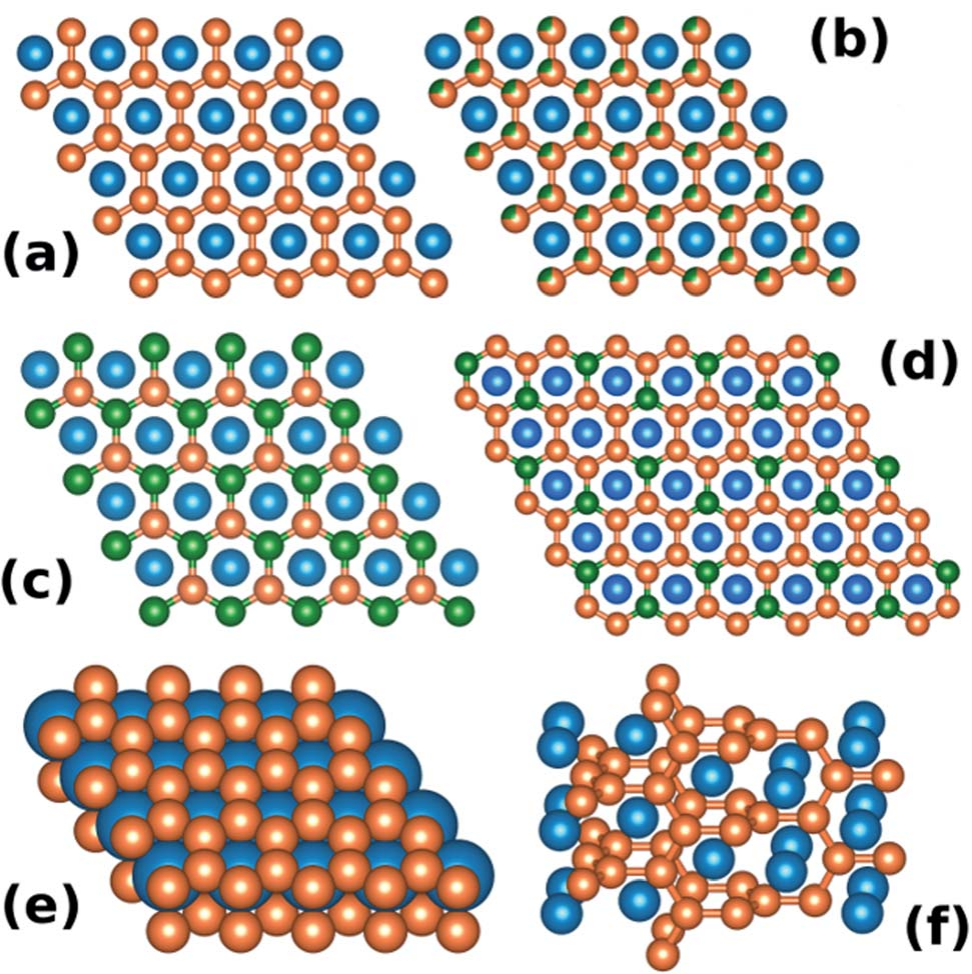
(a) View of the AlB_2_-type structure along the *c* crystallographic axis. Atoms occupying the 2d Wyckoff site (orange) form a honeycomb lattice. (b) Disordered ternary variant of AlB_2_. (c) KZnAs-type ordered ternary variant, (d) Ce_2_CoSi_3_-type ordered ternary variant. Panel (e) shows the AlB_2_-type as a dense-packed lattice of stacked hexagonal and honeycomb layers. Panel (f) presents the α-ThSi_2_ structure, which can be viewed as a “twisted” AlB_2_.

The hexagonal AlB_2_-type (space group *P*6/*mmm*, no. 191) is one of the simplest binary structure types. It consists of alternating hexagonal and honeycomb (graphite-like) layers formed by atoms occupying the 1a and 2d Wyckoff sites, respectively (see Fig. 1(a) and (e)). About 200 individual compounds^1^ are reported to crystallize in the AlB_2_-type structure, with more than 1500 intermetallics belonging to one of the 46 types that are related to the AlB_2_ aristotype.^2^Fig. 1(b–d) shows sample ternary variants of the AlB_2_-type structure.

Interesting physical properties are observed in many AlB_2_-type intermetallics, with the most notable example being high-*T*_c_ superconductivity (*T*_c_ = 39 K) in MgB_2_. Among the ternary compounds, Y_2_PdGe_3_, Y_2_PtGe_3_, and La_2_NiGe_3_ show superconductivity with *T*_c_ = 3.0, 3.3, and 0.45 K, respectively.^3–5^ Many members of the RE_2_TX_3_ family exhibit spin-glass-like behaviour *e.g.* Tm_2_Ni_0.93_Si_2.94_,^6^ Tb_2_Pd_1.25_Ge_3_, Dy_2_Pd_1.25_Ge_2.75_,^7^ Nd_2_PtGe_3_ (ref. 8) and Gd_2_NiSi_3_.^9^ However, Gd_2_CuGe_3_ is reported as an antiferromagnetic compound with the Neel temperature *T*_N_ = 12 K.^10^ Another example worth noting is Pr_2_NiGe_3_, which shows two subsequent magnetic transitions at *T* = 12 K and *T* = 5.5 K.^5^

Two general factors are important for the stability of the AlB_2_-type structure: the relative sizes of atoms at the 1a and 2d sites and the valence electron count. As discussed by Chen *et al.*,^11^ the ratio of the unit cell parameters *c*/*a*, defined by the relative atomic radii of the constituent elements, governs the packing efficiency in the AlB_2_-type structure. For *c*/*a* < 1.074 the honeycomb layer is compressed due to loose packing within the hexagonal layer, and for *c*/*a* > 1.074 the honeycomb is stretched.

The importance of the valence electron count was illustrated in the case of the dimorphic ThSi_2_ compound by Zheng and Hoffmann.^12^ Depending on the parameters of the synthesis procedure (most notably the temperature) the ThSi_2_ can crystallize in one of the two structurally related allotropic variants: the tetragonal α-ThSi_2_ (Fig. 1(f)) or the AlB_2_-type hexagonal β-ThSi_2_ (Fig. 1(a and e)). As Zheng and Hoffmann show, the tetragonal, “twisted” α structure (see Fig. 1(e)) is stable for the electron count of >4.5 e^−^ per Si atom.^12^ In case of ThSi_2_ the formal electron count is 6 (Th^4+^(Si_2_)^4−^) and in fact the β variant is found to be metastable.

Compounds within the ternary RE_2_TX_3_ family (RE – rare earth metal, T – transition metal, X – Si and Ge), which has been a subject of extensive research interest (see *e.g.* ref. 7, 9 and 13–19 and references therein), usually crystallize either in the disordered hexagonal AlB_2_-type or a variant of the tetragonal α-ThSi_2_ structure, depending both on the composition and preparation method.

Here we report the synthesis of a AlB_2_-type compound Ho_2_Pd_1.3_Ge_2.7_ which has previously been suggested to form in the Ho–Pd–Ge system.^20^ The phase is found to show spin-glass behaviour due to the presence of geometrical frustration and crystal lattice disorder. Our attempts to synthesize the analogous Er_2_Pd_1+*x*_Ge_3−*x*_ were not successful, yielding a heterogeneous material. We show that the instability of the Er analogue is caused by the geometrical (packing) factors, which also likely exclude the possibility of synthesizing Tm and Lu analogues, at least under ambient pressure.

## Materials and methods

Polycrystalline samples of RE_2_Pd_1+*x*_Ge_3−*x*_ (RE = Ho, Er; *x* = 0–0.35) were prepared by melting appropriate amounts of high purity chemical elements *i.e.* germanium (99.999%, Alfa Aesar), palladium (99.95%, Alfa Aesar) and holmium (99.9%, Onyxmet) or erbium (99.9%, Onyxmet). Due to volatility of rare earth metals, these chemical elements were used in 2% molar excess. Samples were synthetized in arc furnace (MAM-1 GmbH Edmund Bühler) under purified (Zr-gettered) argon atmosphere. The ingots were re-melted several times by flipping every time to improve reaction among the constituent and promote volume homogeneity. Weight losses after melting process did not exceed 0.5%. Powder X-ray diffraction (pXRD) experiments were performed at room temperature on the powdered as-cast samples using Cu K_α_ radiation on Bruker D2 Phaser diffractometer equipped with XE-T detector. The crystal structure and phase purity were checked by Le Bail analysis of pXRD data using the FullProf software.^13^ Magnetic measurements were performed in temperature range 1.9–300 K in magnetic fields up to 9 T using a Quantum Design Physical Properties Measurement System (PPMS) equipped with AC Measurement System (ACMS). Magnetization measurements were performed for various magnetic fields after field cooling (FC) as well as zero field cooling (ZFC). The heat capacity data were collected in PPMS system by using standard thermal relaxation technique in the temperature range 1.9 K < *T* < 300 K.

Electronic structure calculations were performed on a hypothetical nonmagnetic analogue Y_2_Pd_1+*x*_Ge_3−*x*_ by means of the density functional theory. The Korringa–Kohn–Rostoker method combined with the coherent potential approximation (KKR-CPA)^21^ was used as implemented in the Munich SPR-KKR 7.7 package and the xband 6.3 graphical user interface.

## Results and discussion

**Fig. 2:**
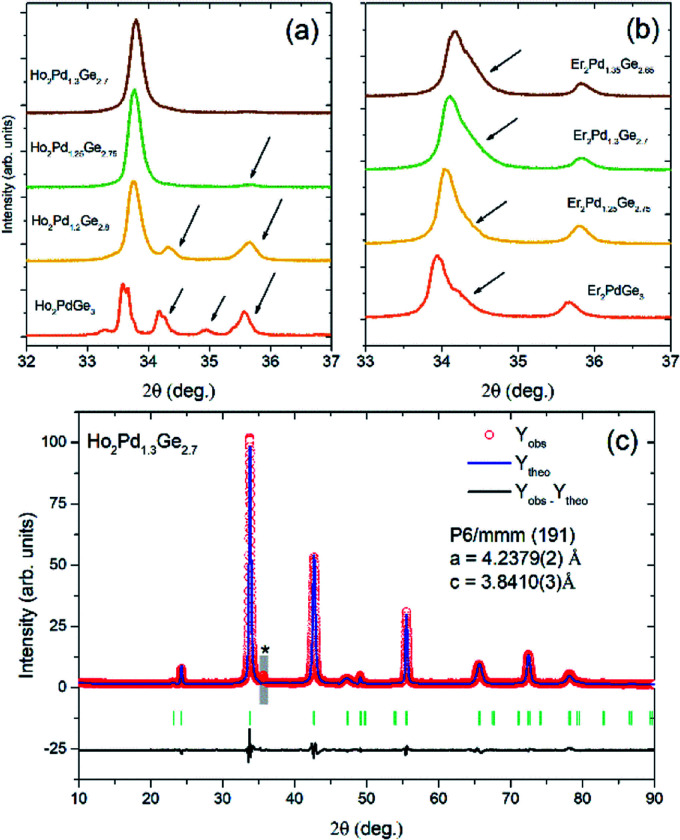
(a and b) pXRD patterns for RE_2_Pd_1+*x*_Ge_3−*x*_ (RE = Ho and Er, respectively) samples with varying Pd : Ge ratio. In both patterns the strongest reflection, appearing around 2*Θ* = 34° is (101). In case of Ho_2_Pd_1+*x*_Ge_3−*x*_ (a) an almost perfectly phase pure sample is obtained for *x* = 0.3 with a small, broad impurity peak appearing at 2*Θ* ≈ 35.5° (see also panel (c) where the peak is more pronounced due to a different scale and is marked with an asterisk). For lower Pd content the samples are contaminated with significant amounts of secondary phase(s). In the Er case, the main reflection is strongly asymmetric, and for the lowest Pd content splits into two broad peaks, suggesting sample inhomogeneity. (c) Rietveld fit to the diffraction pattern of Ho_2_Pd_1.3_Ge_2.7_. A weak impurity phase reflection (barely seen in panel (a)) is marked with an asterisk. The narrow region containing the peak (gray rectangle) was excluded from the refinement.

The room-temperature pXRD patterns for RE_2_Pd_1+*x*_Ge_3−*x*_ samples (Fig. 2) reveal the presence of additional impurity phase for fully stoichiometric Ho_2_PdGe_3_ and Er_2_PdGe_3_. These parasitic phase were identified as HoPd_2_Ge_2_ (ThCr_2_Si_2_-type structure) or ErPdGe_2_ (YIrGe_2_-type structure) and cannot be removed by thermal annealing. However, single-phase compounds can be synthetized by deliberately tweaking the proportion of initial stoichiometry by changing ratio Pd to Ge. Adjusting the Pd : Ge ratio has an effect both on the effective atomic size ratio and the electronic structure, as will be discussed later. No superstructure reflections were observed, in agreement with the assumed disordered AlB_2_-type structure.

The highest phase purity was obtained for *x* = 0.3 in Ho_2_Pd_1.3_Ge_2.7_. In case of Er_2_Pd_1.3_Ge_2.7_, the Bragg peaks were found to be strongly asymmetric, suggesting distortion from the AlB_2_-type structure and/or sample inhomogeneity. Similar effect is observed *e.g.* in solid solutions above the solubility limit. The only impurity peak observed in Ho_2_Pd_1.3_Ge_2.7_ appears at 2*Θ* ≈ 35.5° and likely arises from a trace amount of a tetragonal ternary Er–Pd–Ge ThSi_2_-type phase.

In Ho_2_Pd_1.3_G_2.7_ the 00*l* reflections are much more broadened than others, which suggest a presence of stacking faults in the structure, the anisotropic strain distribution, or a combination of both. In order to improve the Rietveld fit, the effect was modelled using the quartic model of anisotropic strain implemented in FullProf.^22,23^ A similar anisotropic broadening was also observed in Tb_2_Pd_1+*x*_Ge_3−*x*_ and Dy_2_Pd_1+*x*_Ge_3−*x*_.^7^

Results of the Rietveld refinement are presented in Table S1 of the ESI.† The *c*/*a* ratio derived from the Rietveld fit for Ho_2_Pd_1.3_Ge_2.7_ is 0.91, suggesting that the PdGe_3_ honeycomb network is under a compressive strain. Reported cell parameters of RE_2_PdX_3_ (RE = rare-earth elements, X = Si, Ge) compounds are gathered in Table 1. The estimated *c*/*a* ≈ 0.88 for Er_2_Pd_1+*x*_Ge_2−*x*_ phase is lower than any of the reported values, suggesting the strongest compression within the *ab* plane leading to structural instability.

**Table 1:** Unit cell parameters for the reported RE_2_TGe_3_ compounds (RE – rare earth metals, T – Ni, Pd, Pt). Ho_2_Pd_1.3_Ge_2.7_ (highlighted in bold) shows the lowest value of *c*/*a* among the reported ternary disordered AlB_2_-type phases. The estimated ratio for Er analogue (italic) is even lower, resulting in structural instability

Compound	*a* (Å)	*c* (Å)	*c*/*a*
**Ni-bearing**			
Y_2_NiGe_3_ (ref. 24)	4.066	4.025	0.99
La_2_NiGe_3_ (ref. 24)	4.180	4.334	1.04
Ce_2_NiGe_3_ (ref. 25)	4.164	4.243	1.02
Nd_2_NiGe_3_ (ref. 26)	4.145	4.182	1.01
Gd_2_NiGe_3_ (ref. 5)	4.085	4.119	1.01
Ho_2_NiGe_3_ (ref. 5)	4.060	3.970	0.98

**Pd-bearing**			
Y_2_PdGe_3_ (ref. 27)	4.192	4.000	0.95
Nd_2_PdGe_3_ (ref. 27)	4.241	4.193	0.99
Gd_2_PdGe_3_ (ref. 28)	4.215	4.049	0.96
Tb_2_Pd_1.25_Ge_2.75_ (ref. 7)	4.229	3.943	0.93
Dy_2_Pd_1.25_Ge_2.75_ (ref. 7)	4.230	3.944	0.93
**Ho**_**2**_ **Pd**_**1.3**_ **Ge**_**2.7**_	**4.238**	**3.841**	**0.91**
*Er*_*2*_ *Pd*_*1.3*_ *Ge*_*2.7*_	*4.256*	*3.732*	*0.88*

**Pt-bearing**			
Y_2_PtGe_3_ (ref. 4)	4.196	3.994	0.95
Nd_2_PtGe_3_ (ref. 8)	4.246	4.193	0.99

The stabilizing effect of adjusting the Pd : Ge ratio may stem from combination of a number of factors, including (a) change in the effective relative atomic radii of the honeycomb and hexagonal layer atoms, (b) change in the electronic structure, as introducing additional Pd atoms changes the valence electron count, and (c) entropic stabilization.

The latter is rather a subtle effect in case of Ho_2_Pd_1+*x*_Ge_3−*x*_ as the relative difference in mixing entropy 
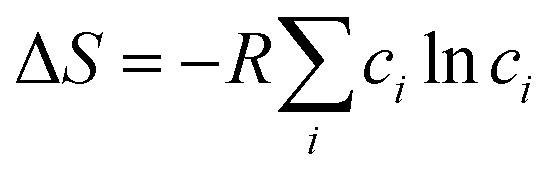
 (with *c*_*i*_ standing for fractional atomic concentrations of Pd and Ge) between the 1 : 3 and 1.3 : 2.7 ratio is only *ca.* 12%. This might be, however, an important contribution in case of systems that are on the edge of instability.^29,30^

The problem of estimation of the effective atomic size is rather complicated in the Ho_2_Pd_1+*x*_Ge_3−*x*_. Depending on the type of atomic size measure (atomic, covalent, or ionic radius) and the specific way it is estimated, the relative size of Pd with respect to Ge can vary substantially. If the covalent radii are taken, as tabulated by Cordero *et al.*,^31^ Pd is about 16% larger than Ge (1.39(6) pm *vs.* 1.20(4) pm, respectively). In case of Slater's atomic radii,^32^ Pd is 12% larger than Ge. Since the Pd–Ge honeycomb layer is under a compressive stress in Ho_2_Pd_1+*x*_Ge_3−*x*_, the stabilizing effect of introducing a larger atom is rather surprising, suggesting that the electronic structure factors might be important in this case. It is worth noting, however, that if one considers the electronic density-based atomic radii by Rahm, Hoffmann and Ashcroft,^33,34^ the Pd is smaller by *ca.* 8% than Ge. The atomic size ratio effect is also consistent with the fact that the RE_2_PdGe_3_ compounds with larger RE lanthanides seem to be stable with the nominal Pd : Ge ratio 1 : 3.

The results of electronic structure calculations on a hypothetical Y_2_Pd_1+*x*_Ge_3−*x*_ system with lattice parameters set to the values obtained for Ho_2_Pd_1.3_Ge_2.7_, show that the introduction of additional Pd only weakly affects the electronic density of states (Fig. S1 of the ESI†) and band structure in a manner that cannot be easily described within a rigid band approximation (simple shifting of the Fermi level *E*_F_ with changing the number of valence electrons). The DOS peak centered at around −3 eV is slightly broadened and increased in height in case of “Y_2_Pd_1.3_Ge_2.7_” compared to the nominal 1 : 3 stoichiometry.

Thus, the stabilizing effect of increased Pd cannot be simply ascribed to any particular factor. Moreover, since the samples are synthesized by arc-melting the constituent elements, changing of the Pd : Ge ratio may also influence the synthetic conditions (the liquidus temperature of 13 : 27 Pd–Ge alloy is about 60 °C lower than of 1 : 3,^35^ resulting in an easier melting of the metallic button).

**Fig. 3:**
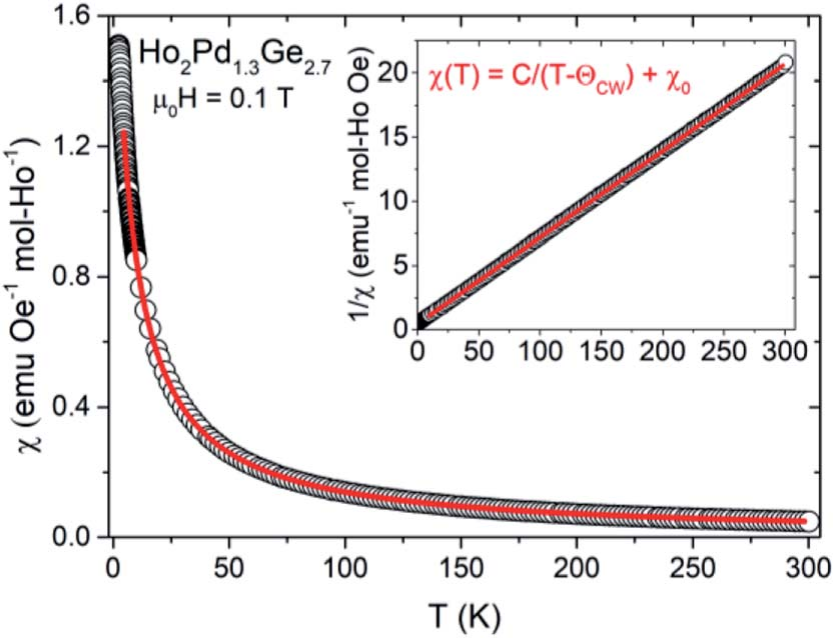
The temperature dependence of the magnetic susceptibility for Ho_2_Pd_1.3_Ge_2.7_. The red line is fit to Curie–Weiss law for temperatures above 15 K. The inset shows inverse magnetic susceptibility in function of temperature with fitted function 1/*χ* = *T*/*C* − *θ*_CW_/*C* (red line).

The DC magnetic susceptibility (*χ* = *M*/*H*) as a function of temperature were made under an applied magnetic field 0.01, 0.1 and 1 T for Ho_2_Pd_1.3_Ge_2.7_ As shown in Fig. 3, *χ*(*T*) increases with decreasing temperature, which is typical behavior for Curie–Weiss paramagnets. This dependence can be described by the formula:*χ* = *χ*_0_ + *C*/(*T* − *θ*_CW_)where *χ*_0_ is the temperature-independent susceptibility, *C* means the Curie constant and *θ*_CW_ is the paramagnetic Curie temperature. Insets of Fig. 3 exhibit plots of inverse magnetic susceptibility *versus* temperature, which are linear in the range *T* = 15–300 K for Ho_2_Pd_1.3_Ge_2.7_. The Curie–Weiss law was fitted to experimental data in linear region and obtained values of *θ*_CW_ and *C* are gathered in Table 2. Negative value of *θ*_CW_ suggests antiferromagnetic coupling between the magnetic moments. The effective magnetic moment was calculated using equation:
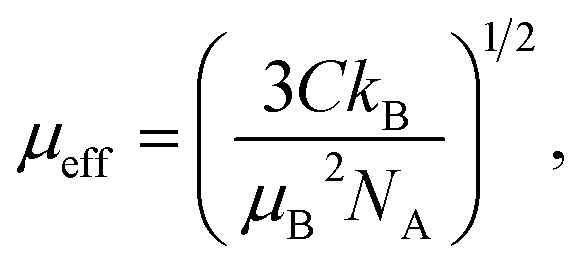
where *k*_B_ is the Boltzmann constant, *μ*_B_ is the Bohr magneton and *N*_A_ is the Avogadro number. Obtained values are found to close to the theoretical free ion value of Ho^3+^.

**Table 2:** Selected physical properties data for Ho_2_Pd_1.3_Ge_2.7_

Freezing temp., *T*_f_ (K)	2.3
Frustration index, *f*	3.3
Weiss temp., *θ*_CW_ (K)	−7.5
Effective magnetic moment, *μ*_eff_ (*μ*_B_)	10.94
Activation energy, *E*_a_/*k*_B_ (K)	23
*T*_0_ (K)	1.5
Tholence parameter, *δT*_Th_	0.32
*M*_0_ (emu g^−1^)	0.44
Magnetic viscosity, *S* (emu g^−1^)	0.0024

**Fig. 4:**
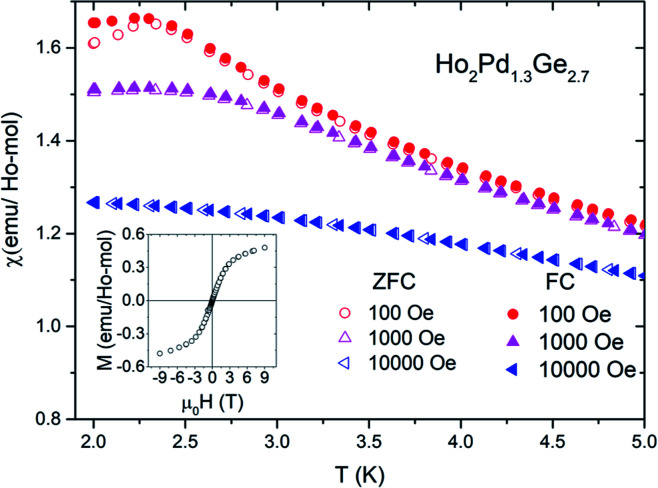
The difference between ZFC and FC magnetic susceptibility of Ho_2_Pd_1.3_Ge_2.7_ at different applied magnetic field. Insets shows *M*(*H*) curves for Ho_2_Pd_1.3_Ge_2.7_ at *T* = 2 K.

Results of low-temperature *χ*_DC_(*T*) measurements in zero field cooling (ZFC) as well as field cooling (FC) conditions for different applied magnetic fields are shown in Fig. 4. For Ho_2_Pd_1.3_Ge_2.7_ in both measurement modes a maxima is observed around *T* = 2 K. Low field (100 Oe) curves deviate below this temperature, which suggests the presence of irreversibility associated with magnetic metastability in this compound.^12^ The transition temperature *T*_T_ = 2.3 K (defined as the position of the maximum of d(*χT*)/d*T*). Moreover *T*_T_ is strongly suppressed by an applied magnetic field. The transition temperature is over three times smaller than |*θ*_CW_|. The empirical measure of frustration, defined as *f* = |*θ*_CW_|/*T*_T_, is *f* = 3.3, which implies strong magnetic frustration and suggests formation of a glassy state rather than an antiferromagnetic order. This hypothesis may be confirmed by the field dependence of isothermal (*T* = 2 K) magnetization depicted in the inset of Fig. 4. No magnetic hysteresis is observed down to 2 K and a “S”-shape behavior of *M*(*H*) can be seen. Despite the application of strong magnetic field (*μ*_0_*H* = 9 T) the magnetization curves do not saturate, which is probably due to lack of long-range magnetic ordering. The same phenomenon was observed in case Tb_2_Pd_1.25_Ge_2.75_ and Dy_2_Pd_1.25_Ge_2.75_.^9^ For that reason a peak of magnetization around *T*_T_ may be interpreted as a result of spin-freezing transition.

**Fig. 5:**
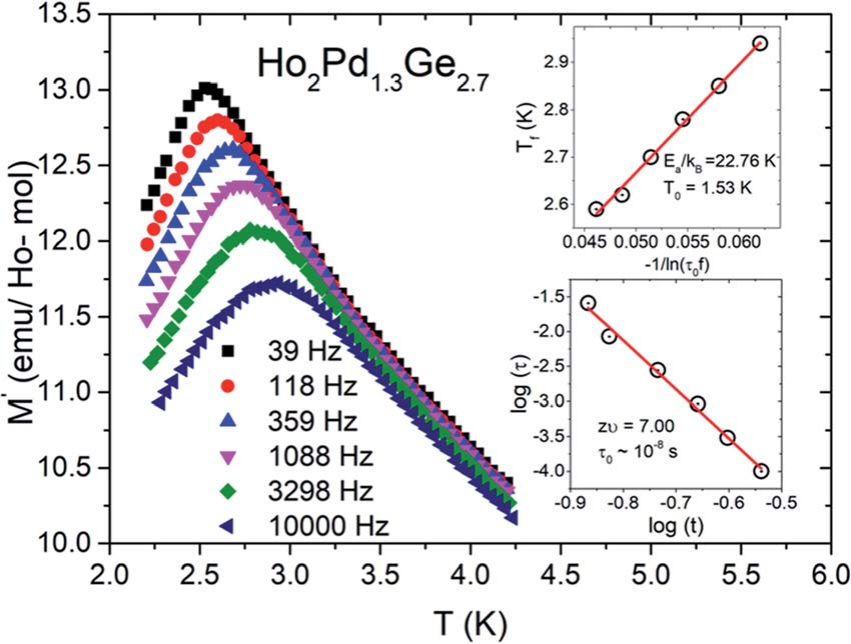
Temperature dependence of the real part of the AC magnetic susceptibility *χ*(*T*) for Ho_2_Pd_1.3_Ge_2.7_. The inset (I) shows ln(*τ*) plotted as a function of ln(*t*) with the solid red line, which represents the fit to the power-law divergence. The inset (II) shows plot of the freezing temperature (*T*_f_) *versus* 1/ln(*τ*_0_*f*) with a Vogel–Fulcher law fit (red solid line).

To confirm spin/cluster-glass behavior, AC susceptibility measurements were carried out in an excitation field 3 Oe for frequencies *ν* = 39, 118, 359, 1088, 3298 and 10 000 Hz (logarithmic spacing). The real part of AC magnetization (*M*′) as a function of temperature for various frequencies is plotted for Ho_2_Pd_1.3_Ge_2.7_ in Fig. 5. The maximum of *M*′ appears close to the irreversibility temperature obtained from *χ*_DC_(*T*). Moreover this peak is very sensitive to the applied frequency and shifts to higher temperature region with increase in AC frequency, which reveals glassy formation in this compound.^7,12,16,17^ In a typical glassy systems the relative shift in freezing temperature can be described by the *δT*_f_ parameter:^7^
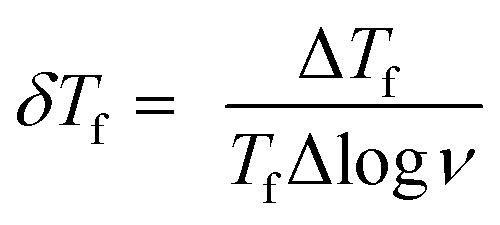
where *T*_f_ is a spin freezing temperature for the lowest measured frequency. This temperature is usually defined as a the calculated value of *δT*_f_ = 0.04 for Ho_2_Pd_1.3_Ge_2.7_ is one order of magnitude larger than those reported for canonical spin-glasses,^7,12^ however it lies in a range that is typical for cluster-glass.^7,17^

For a glassy systems spin freezing state can be modeled by the power law:^7,8^
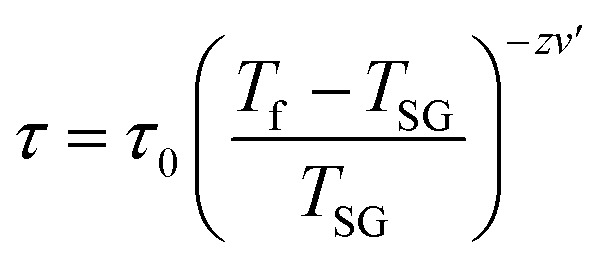
where *τ* means the relaxation time associated with the measured frequency (*τ* = 1/*ν*), *zv*′ is dynamic critical exponent, *τ*_0_ is microscopic single spin flipping time, *T*_SG_ is spin-glass temperature in static limit (*ν* → 0). To simplicity, we have determined the value of *T*_SG_ to be equal *T*_T_. The value of *τ*_0_ and *zv*′ have been estimated from linear fit of log(*τ*)–log(*t*) dependence, which is shown in the inset (I) of Fig. 5 and are within the characteristic range reported for typical cluster-glass systems.^7^

The second plausible interpretation of the freezing process can be expressed by empirical Vogel–Fulcher law:^16,17^
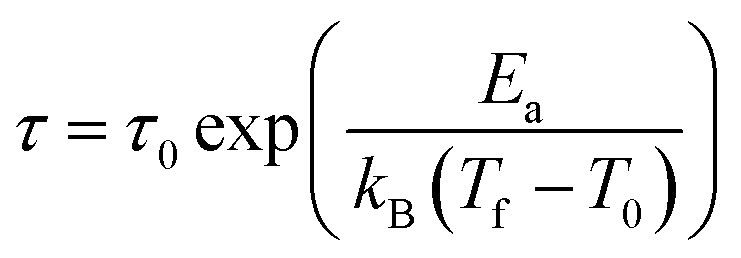
where the fitting parameters *T*_0_ and *E*_a_ are known as Vogel–Fulcher temperature and activation respectively. In this formula *k*_B_ is the Boltzmann constant. The above equation may be rewrite as a simple relation between *T*_f_ and *ν*:
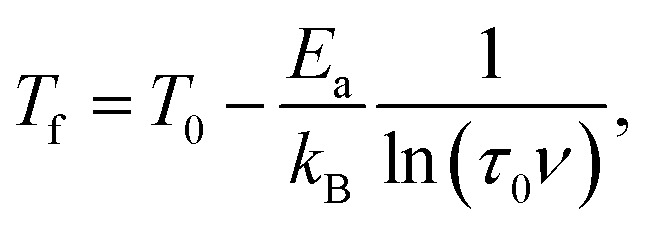
which is fitted to data shown in the inset of Fig. 5. The best estimated values of *T*_0_ and *E*_a_ for Ho_2_Pd_1.3_Ge_2.7_ are gathered in Table 2. The *E*_a_/(*k*_B_*T*_0_) ratio is much greater than 1, which indicates a weak coupling between the magnetic entities and is typically observed for cluster-glass systems.^36^ Another evidence of formation spin-glass like state in Ho_2_Pd_1.3_Ge_2.7_ is Tholence criterion:^37^*δT*_Th_ = (*T*_f_ − *T*_0_)/*T*_f_ = 0.32, which is in agreement with the cluster glass scenario.

The time evolution of magnetization resulting from slow relaxation is shown in Fig. S2 in ESI† and was studied through ZFC measurements at different temperatures (*T* = 1.8, 2.5 and 5 K). Samples were cooled under zero applied magnetic field to required temperature and after waiting for a certain time small amount of magnetic field (*H*_dc_ = 1000 Oe) was applied to measure the time dependence of magnetization. This phenomenon, called also an ageing effect, is caused by non-equilibrium dynamical state below freezing temperature and is characteristic behavior for different types of glassy systems.^7,12,19–21^ The relaxation process are commonly described by equation: *M*(*t*) = *M*_0_ + *S* ln(*t*/*t*_0_ + 1), where *M*_0_ is magnetization at *t* = 0 and *S* is the magnetic viscosity.^20,21^ Both these parameters are depending on temperature in contrast to *t*_0_, which depends on the measuring conditions and have only limited physical relevance and is typically orders of magnitude larger than intrinsic relaxation time.^21^ The red solid line in Fig. S2† show the best fitting results obtained by the last-squares method. Estimated values of *M*_0_ and *S* for compounds with Ho and Er are collected in Table 2 and are in good agreement with data found in literature.^19–21^ It is worth to note that the absence of magnetic relaxation behavior for measurements above *T*_f_ is expected as the aging effect exist only in glassy-state.

The main panel of Fig. S3 of ESI† depicts zero-field heat capacity *C*_p_ as a function of temperature for Ho_2_Pd_1.3_Ge_2.7_ The *C*_p_ saturates value at room temperature near the classical Dulong–Petit value, *C*_p_ = 3*nR* ≈ 150 J mol^−1^ K^−1^, where *n* is the number of atoms per formula unit (*n* = 6) and *R* is the gas constant (*R* = 8.314 J mol^−1^ K^−1^). The inset of Fig. S2† exhibit dependence of *C*_p_/*T* at low temperatures. The broad peak around *T*_f_ can indicate a magnetic phase transition and is in a good agreement with magnetization measurements data. The shape of this peak may suggest the absence of long range antiferromagnetic order and is similar to that observed for other glassy magnetic materials.^7–9,12^

Magnetization and heat capacity results suggest a glassy transition in contrast to long-range AFM order observed in Ho_2_PdSi_3_ single crystals.^38^ The difference of magnetic properties between Ge- and Si-bearing compound likely stems from the higher degree of lattice disorder present in the former which manifests itself in rather broad XRD peaks with strong anisotropic broadening found in Ho_2_Pd_1.3_Ge_2.7_. In Ho_2_PdSi_3_ single crystals the presence of a long-range superstructure along the *c* direction is possible, as it was observed in Gd_2_PdSi_3_.^39^

## Conclusions

A polycrystalline sample of a new AlB_2_-type intermetallic compound Ho_2_Pd_1.3_Ge_2.7_ was synthesized by an arc-melting technique. We have shown that the phase stability is improved by tuning the Pd : Ga ratio out of the ideal 1 : 3.

Attempts to synthesize an Er analogue resulted in heterogeneous materials. Its instability can be understood in terms of atomic packing factor. Obtained magnetization and heat capacity results suggest that Ho_2_Pd_1.3_Ge_2.7_ exhibits a spin-glass like behavior with freezing temperature *T*_f_ = 2.3 K.

## Conflicts of interest

There are no conflicts to declare.

## Supplementary Material

RA-011-D1RA04422B-s001

RA-011-D1RA04422B-s002
