# Supplementary material for: Ho2Pd1.3Ge2.7 – a ternary AlB2-type cluster glass system
Source: RSC Adv. 2021 Jul 21;11(41):25187–93. doi: 10.1039/d1ra04422b (PMC9036949; doi:10.1039/d1ra04422b)
Supplement: RA-011-D1RA04422B-s001 [file RA-011-D1RA04422B-s001.pdf]

Electronic Supplementary Material (ESI) for RSC Advances.
This journal is © The Royal Society of Chemistry 2021

$Ho_2Pd_{1.3}Ge_{2.7}$ – a ternary AlB_2-type cluster glass system

Leszek S. Litzbarski,*[a] Tomasz Klimczuk [a] and Michał J. Winiarski [a]

[a] *Faculty of Applied Physics and Mathematics and Advanced Materials Center, Gdansk University of Technology, Narutowicza 11/12, 80-232 Gdansk, Poland*

* Corresponding author: leszek.litzbarski@pg.edu.pl

Supplementary Material

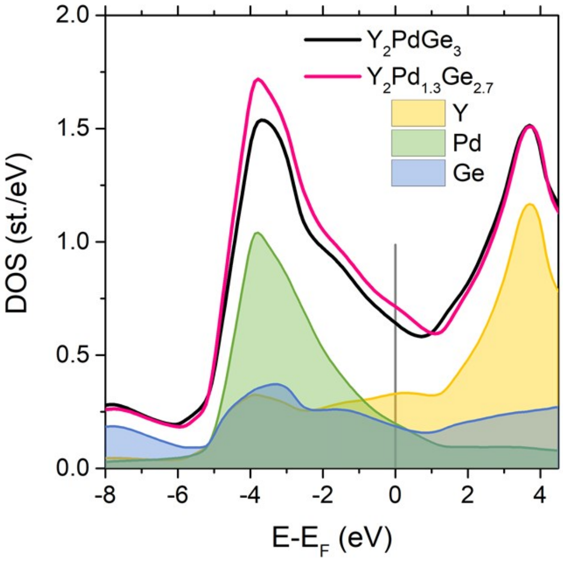

Figure S1: Electronic density of states calculated for $Y_2Pd_{1+x}Ge_{3-x}$ with x = 0 (black line) and 0.3 (pink

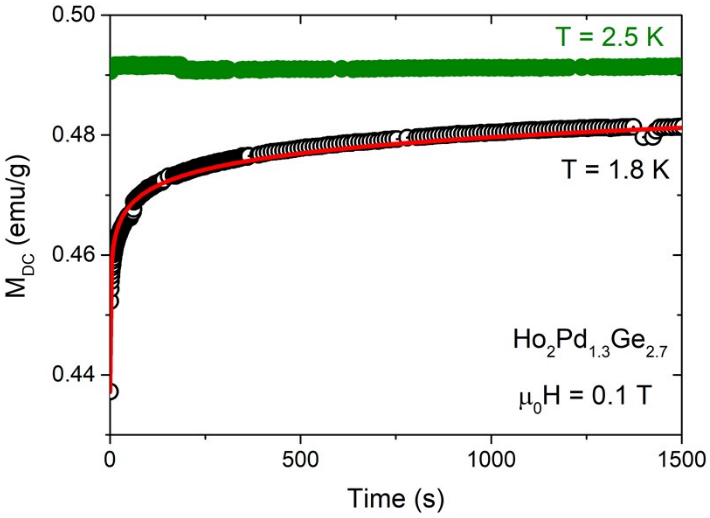

line). Substitution of Ge with additional Pd increases slightly the DOS around the Fermi level. The overall shape of the DOS is found to be consistent with a previous report by Sampathkumaran *et al.* [1]

Figure S2 Time dependent remnant magnetization behavior for $Ho_2Pd_{1.3}Ge_{2.7}$. Solid red line represents a fit to equation $M(t) = M_0 + S\,ln(t/t_0 + 1)$.

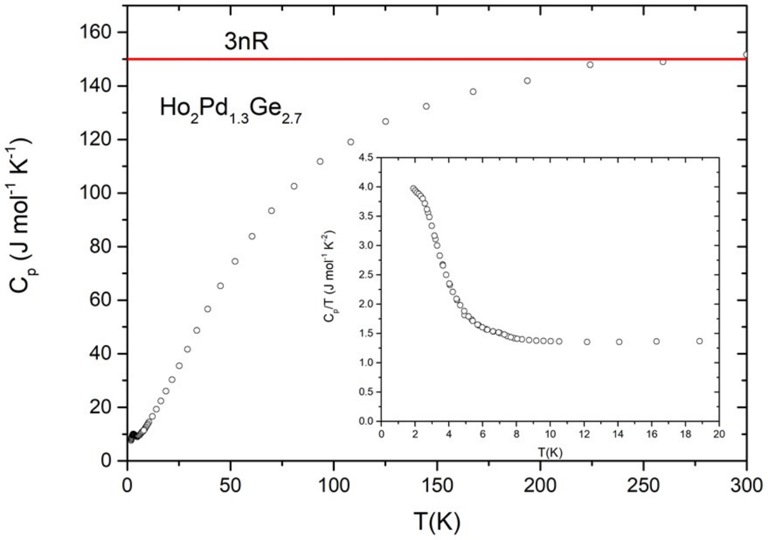

Fig. S3 Temperature dependence of the heat capacity (C_p) for $Ho_2Pd_{1.3}Ge_{2.7}$. Insets shows a plot of C_p/T vs. T at low temperatures measured without applied magnetic field.

Table S1. Results of Rietveld refinement on PXRD data for $Ho_2Pd_{1.3}Ge_{2.7}$

Fit reliability factors				
R_p	10.2%	R_{exp}		4.26%
R_{wp}	10.8%	χ^2		6.45
Unit cell parameters (Å):				
a =	4.2379(2)	*c* =		3.8410(3)
Atomic parameters				
**Atom**	**x**	**y**	**z**	**B_{iso} (Å^2)**
Ho	0	0	0	1.87(4)
Pd (occ. 0.325) Ge (occ. 0.675)	⅓	⅔	½	1.18(4)

References:

1 E. V. Sampathkumaran, S. Majumdar, W. Schneider, S. L. Molodtsov and C. Laubschat, Superconductivity in Y2PdGe3, *Phys. B Condens. Matter*, 2002, **312–313**, 152–154.
